# Improving timely treatment with a stroke emergency map: The case of northern China

**DOI:** 10.1002/brb3.1743

**Published:** 2020-07-11

**Authors:** Tianli Zhang, Xiaodong Zhang, Huisheng Sun, Feng Zhou, Shiqin Lin, Hui Sang, Nannan Zheng, Ziyi Zhao, Jing Shi, Weirong Li

**Affiliations:** ^1^ Department of Neurology Taiyuan Central Hospital of Shanxi Medical University Taiyuan China; ^2^ Administration office Taiyuan Health Commission Taiyuan China; ^3^ Department of Neurology Changzhi Medical College Affiliated Heping Hospital Changzhi China; ^4^ Medical Records Statistics Office Shanxi Bethune Hospital Taiyuan China

**Keywords:** acute ischemic stroke, functional outcome, intravenous thrombolysis, prehospital delay, stroke emergency map

## Abstract

**Objective:**

The Chinese stroke emergency map (*SEM*) was implemented in 2017 to reduce prehospital and hospital delays for acute ischemic stroke (AIS) patients suitable for intravenous recombinant tissue plasminogen activator (rt‐PA) thrombolysis. However, data on the time delay following the implementation of an *SEM* in China are limited.

**Methods:**

Data for suspected stroke patients from the *SEM* registry center of Taiyuan, Shanxi Province, from August 2017 to July 2019, patients’ characteristics, thrombolysis rate, and functional outcome at 90 days were analyzed.

**Results:**

One thousand seven hundred and eighty six patients who arrived at hospitals within 4.5 hr of onset were included; 35.9% arrived by emergency medical services (EMSs), and 1,207 (67.6%) of the population received intravenous rt‐PA. As a result of the *SEM*, the number of patients treated with rt‐PA increased from 63.9% in phase 1 (August 2017 to July 2018) to 70.5% in phase 2 (August 2018 to July 2019). The median onset‐to‐door and onset‐to‐needle times decreased by five minutes (100 [IQR: 62–135] vs. 105 [IQR: 70–145], *p* = .005) and nine minutes (158 [IQR: 124–197] vs. 167 [IQR: 132–214], *p* = .001), respectively. Patients in phase 2 achieved greater independent function outcome at 90 days (79.9% vs. 72.1%; adjusted odds ratio, 2.010; 95% confidence interval, 1.444–2.798). The binary logistic regression models revealed that shorter onset‐to‐needle time (OR: 0.994; 95% CI: 0.992–0.997; *p* < .001) and lower baseline NIHSS scores (OR: 39.120; 95% CI: 23.477–65.188; *p* < .001 and OR: 18.324; 95% CI: 11.425–29.388; *p* < .001 and OR: 3.123; 95% CI: 2.044–4.773; *p* < .001) were significant predictors for the independent function outcome.

**Conclusion:**

The implementation of a stroke emergency map is more likely to reduce prehospital delays and improve function outcomes. Future efforts should attempt to increase EMS usage.

## Graphical abstract

The implementation of a stroke emergency map effectively reduced prehospital delays and improved function outcomes. Future efforts should attempt to increase EMS usage.

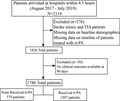

## 1 INTRODUCTION

Stroke is the leading cause of adult death and disability in China (Wang, Abajobir, et al., 2017). With the continuous promotion of stroke care and prevention, the standardized mortality rate shows a downward trend in China (Wang, Hu, Sang, Luo, & Yu, 2017), while there is a significant upward trend for disability‐adjusted life years (DALY; Report on Stroke Prevention & Treatment in China, 2018) Early reperfusion therapy, including intravenous recombinant tissue plasminogen activator (rt‐PA) thrombolysis within 4.5 hr and mechanical thrombectomy (MT) within six hours of symptom onset, is the most effective evidence‐based treatment for acute ischemic stroke (AIS; Powers et al., 2018). The outcomes for thrombolysis guided by perfusion imaging up to nine hours (Ma et al., 2019) and MT for selected patients up to 24 hr (Albers et al., 2018; Nogueira et al., 2018) after the onset of stroke are extremely time‐dependent (Wardlaw, Murray, Berge, & del Zoppo, 2014). Previous research indicates that for each minute earlier that treatment is initiated, there are up to 12.5 fewer disability‐adjusted days (Meretoja et al., 2014; Meretoja, Keshtkaran, Tatlisumak, Donnan, & Churilov, 2017; Schlemm, 2018). However, in China, only about 10%–20% of patients arrive at hospitals within three hours, and less than 3% of patients with AIS receive rt‐PA (Wu et al., 2019). A poor understanding and ability to identify stroke symptoms, prehospital delays, and nonstandardized emergency workflows are the top three factors for delays in timely treatment (Jiang et al., 2016; Pulvers & Watson, 2017). To improve rapid access to rt‐PA, the first stroke emergency map (*SEM*) was launched in Shenzhen in November 2016, and as a result, the rate of patients receiving rt‐PA thrombolysis and endovascular thrombectomy has increased significantly (Ye et al., 2019). Subsequently, the *SEM* was designed to increase the likelihood that treatment would occur in the golden first‐aid window of “three one hour” (no more than one hour from onset to call for ambulance, no more than one hour from prehospital transfer, and no more than one hour from admission to thrombolysis) and was released in many cities by Chinese healthcare administrations.

Stroke incidence and mortality rates are highest in the northeast, the lowest in the south, and prominent in the middle of China (Report on Stroke Prevention & Treatment in China, 2018; Wang, Jiang, et al., 2017). The Shanxi Province is located in Loess Plateau, the north area of China, where economic development and medical technology are currently lagging behind the eastern and coastal regions. In this region, 274.74 new strokes and 56.9 stroke‐related deaths occurred per 100,000 individuals in 2016. An epidemiological survey of stroke patients in the Shanxi province involved 714,893 individuals (aged ≥ 40 years) and revealed that Taiyuan had the highest proportion of stroke hospitalization (3.61%) (Luo, Jiang, Yu, Luo, & Nan, 2019). Based on that survey, a stroke emergency map was developed in July 2017 in Taiyuan, as part of a rapid regional stroke treatment system. This study evaluates the time delays and functional prognosis of patients who received rt‐PA thrombolysis over the two years following the implementation of the *SEM*. The purpose of this approach is to facilitate optimization of the stroke‐related health service system and provide more stroke patients with the best opportunity to arrive at hospitals within the optimal time frame to maximize patients’ prognosis.

## 2 METHODS

### 2.1 Patient selection

**Figure 1:**
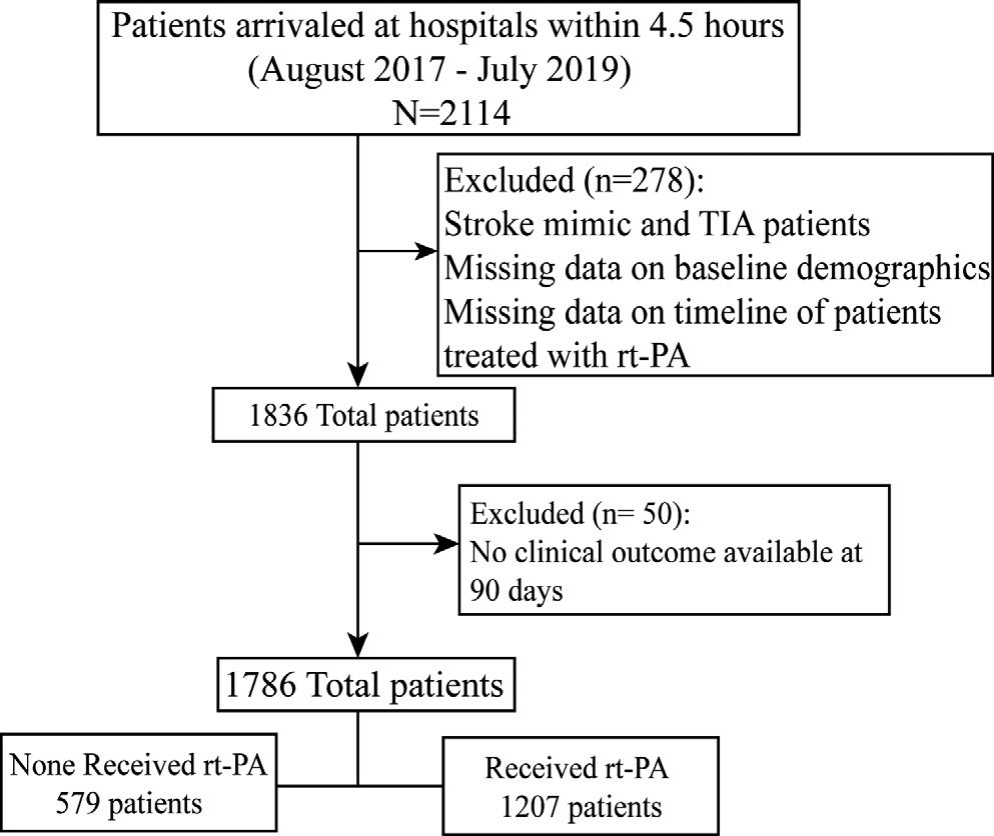
Overview of patients included. rt‐PA, recombinant tissue plasminogen activator; TIA, transient ischemic attack

Data for 2,114 consecutive suspected stroke patients acquired from the *SEM* registry who arrived at 19 hospitals within a 4.5‐hr time window were collected between August 2017 and July 2019. We excluded stroke mimic and TIA patients (*n* = 60) and patients with no information on baseline demographics and timeline if they received rt‐PA thrombolysis (*n* = 218). After the exclusions, an initial cohort of 1836 patients was assembled. To evaluate the functional outcome at 90 days, we further excluded patients with no clinical outcome available at 90 days (*n* = 50). The final analysis sample consisted of 1,786 patients (Figure 1).

This retrospective study was approved by the Institutional Review Board of Taiyuan Central Hospital, Shanxi, China, in accordance with the Declaration of Helsinki.

### 2.2 Data collection

We collected the following baseline characteristics: demographics including age and sex; and vascular risk factors such as hypertension, diabetes mellitus, dyslipidemia, coronary heart disease, atrial fibrillation, and previous history of ischemic stroke. The National Institutes of Health Stroke Scale (NIHSS) score on admission, presence of intracerebral hemorrhages, the mode of transportation (emergency medical service [EMS] use and no EMS use), and the type of stroke‐related service hospital (primary stroke center [PSC] and comprehensive stroke center [CSC]) selected by patients were recorded. Intervals (onset‐to‐door time, door‐to‐needle time, and onset‐to‐needle time) in rt‐PA‐treated patients were also documented.

### 2.3 The details of the *SEM*

#### 2.3.1 Certification of qualified hospitals with intravenous thrombolysis

The Department of Neurology Quality Supervision of Taiyuan investigated rt‐PA thrombolysis in 24 general hospitals with independent neurology in six districts, three counties, and one city. The hospitals with stroke treatment capacity, capability for intravenous thrombolysis, presence of a stroke team with 24/7 on‐call, and where head computed tomography (CT) was available with 24/7 voluntarily participated in the stroke emergency map were certified as qualified hospitals and assorted according to geographical location. Ultimately, 15 hospitals (eight primary stroke centers [PSC] and seven comprehensive stroke centers [CSC]) were selected from August 2017 to July 2018, and four more (all PSC) hospitals were added from August 2018 to July 2019.

#### 2.3.2 Treatment process protocol for patients with stroke

If the suspected stroke patient calls the emergency phone #120 within 4.5 hr of the onset of symptoms, the dispatch command center dispatches an ambulance and emergency personnel nearby according to the symptoms described who typically arrive within ten minutes, completes the medical history collection while in the ambulance, and employs the “face arm speech test” (FAST) to confirm the case of stroke. If a stroke is highly suspected, the following protocol is followed: complete blood glucose, blood oxygen, and electrocardiogram, and clinicians are also expected to keep the airway open, establish venous channels, and then identify whether the large vessel is occluded according to the Field Assessment Stroke Triage for Emergency Destination (FAST‐ED).

If large vessel occlusion was not considered, protocol dictates a transfer to the nearest qualified hospital if it is CSC. If large vessel occlusion is highly suspected and intravenous thrombolysis combined with endovascular treatment is required, patients may be considered directly transferred to CSC. Patients’ and caregivers’ hospital preferences should be considered during the transfer process. The ambulance crew then notifies the receiving hospital before arrival, and the hospital immediately initiates the stroke team and begins the emergency management program. Upon arrival at the hospital, the patient is quickly scheduled for a brain CT examination. During the CT examination, the emergency personnel and the stroke team (including doctors and nurses) complete the patient transfer. If the CT results do not indicate/contraindicate of stroke, the informed consent of the caregivers is obtained as soon as possible, and intravenous thrombolysis is performed in the emergency room or stroke unit, according to the protocol of the specific hospital. If the suspected stroke patient does not arrive via EMS, triage by emergency or outpatient nurses, then the attending physician and nurse immediately complete the initial evaluation and blood sampling, send patients to brain CT examination, and promptly informed the stroke team within five minutes. All hospitals require DNT < 60 min, and if door‐to‐needle time (DNT) >60 min, the reason for the delay must be documented. All patients are treated according to the Chinese guidelines for diagnosis and treatment of the AIS 2014 and Chinese guidelines for the endovascular treatment of acute ischemic stroke 2015 (Chinese Society of Neurology & Chinese Stroke Society, 2014; Chinese Society of Neurology, Chinese Stroke Society, & Neurovascular Intervention Group of Chinese Society of Neurology, 2015).

#### 2.3.3 Quality improvement of the *SEM*

The program for stroke care quality improvement is dedicated to decreasing the time to initiation of thrombolysis. To achieve this goal, education of stroke diagnosis and treatment process is regularly scheduled for EMS operators and multidisciplinary staff, and a feedback system was designed. Additionally, stroke care quality improvement programs conduct dynamic observations of the monthly stroke registration data reported by each stroke center to assist the EMS and stroke teams in the identification of causes of specific delays and devise improvement measures to address these issues. CSC physicians can assist PSC depending on specific situations via remote consultation and site‐specific consultation. Further, additional professional training has been conducted in hospitals that have yet not qualified to perform thrombolysis so that more locations are available, which will shorten patient transport times. Last, an *SEM* is displayed prominently in ambulances and buses, and physicians from qualified hospitals regularly go out into communities to further public understanding and awareness of stroke.

### 2.4 Endpoints and definitions

A modified Rankin Scale (mRS) score was used to evaluate patients’ functional outcome at 90 days. An mRS score of 0 or 1 indicated a favorable functional outcome and an mRS score of 0–2 a dependent clinical outcome. We also recorded symptomatic intracranial hemorrhage (sICH), defined as a deterioration in NIHSS score of ≥4 with a parenchymal hemorrhage type 2 (Yaghi et al., 2017), and all‐cause mortality within 90 days.

### 2.5 Data analysis

We analyzed data for patients who fulfilled the inclusion criteria between the two time periods: “phase 1” (from August 2017 to July 2018) versus “phase 2” (from August 2018 to July 2019). We compared the baseline characteristics of patients, time intervals, and functional outcome at 90 days between the two phases following the *SEM* implementation using the Student's *t* test, chi‐square test, or Mann–Whitney *U* test, as appropriate. Univariable and multivariable binary logistic regression models were used to explore the association between dependent function outcome at 90 days in patients who received rt‐PA before and after adjustment for the following potential confounders: age, sex, NIHSS score on admission, history of hypertension, history of diabetes mellitus, history of dyslipidemia, history of coronary heart disease, history of atrial fibrillation, history of ischemic stroke, the mode of transportation, the type of stroke‐related service hospital, period of presentation, and onset‐to‐needle time. In the initial univariable models, those factors that contributed to the outcome of interest at *p* values <.1 were included in the multivariable models. A two‐tailed *p*‐value of <.05 was defined as statistically significant. We also compared the characteristics of patients with and without EMS usage (Tables S2–S3). All statistical analyses were performed using SPSS V. 25.0 software.

## 3 RESULTS

A total of 1,786 patients from August 2017 to July 2019 met the inclusion criteria. Among them, 17 (1.0%) were in‐hospital stroke patients, 641 (35.9%) were EMS‐transported, and 1,308 (73.2%) were directly transferred to CSC. The total number of stroke patients within the time window increased from 43.9% in phase 1 to 56.1% in phase 2. The baseline characteristics of the patients are listed in Table 1. Four more new hospitals, including 62 (6.2%) patients, were added in phase 2, so we also compared the baseline characteristics within the initial 15 hospitals between phases 1 and 2 (Table S1). Compared with patients in phase 1, patients in phase 2 were more likely to be transferred to PSC and to use EMS transport to receive rt‐PA or endovascular treatment.

**Table 1:** Baseline characteristics of the study population

Characteristic	Total	Phase 1	Phase 2	*p*‐Value
Patients included (%)		784 (43.9)	1,002 (56.1)	
Age (mean ± *SD*)	64.37 ± 12.53	63.97 ± 12.72	64.68 ± 12.38	.235
Male	1,234 (69.1)	546 (69.6)	688 (68.7)	.656
Hypertension	1,087 (60.9)	456 (58.2)	631 (63.0)	.039
Diabetes	437 (24.5)	199 (25.4)	238 (23.8)	.426
Hyperlipidemia	147 (8.2)	74 (9.4)	73 (7.3)	.100
Coronary heart disease	158 (8.8)	61 (7.8)	97 (9.7)	.161
Atrial fibrillation	160 (9.0)	66 (8.4)	94 (9.4)	.480
History of cerebral infarction	244 (13.7)	118 (15.1)	126 (12.6)	.131
Admission NIHSS score (mean ± *SD*)	7.86 ± 7.61	7.63 ± 7.46	8.05 ± 7.72	.242
EMS use	641 (35.9)	254 (32.4)	388 (38.7)	.006
Type of treatment				
Only intravenous rt‐PA	1,098 (61.5)	454 (57.9)	644 (64.3)	.026
Intravenous rt‐PA + EVT	109 (6.1)	47 (6.0)	62 (6.2)	
Only EVT	71 (4.0)	32 (4.1)	39 (3.9)	
Others	508 (28.4)	251 (32.0)	257 (25.6)	
Arrival hospital				
PSC	478 (26.8)	172 (21.9)	306 (30.5)	.000
CSC	1,308 (73.2)	612 (78.1)	696 (69.5)	

Note

The *p*‐values are based on Student's *t* test (normal distribution) and chi‐square test (categorical variables).

Abbreviations: CSC, comprehensive stroke center; EMS, emergency medical service; EVT, endovascular treatment; NIHSS, National Institutes of Health Stroke Scale; PSC, primary stroke center; rt‐PA, recombinant tissue plasminogen activator.

As shown in Table 2, in patients received *rt‐PA,* history of cerebral infarction, the mode of transportation, and the type of arrived hospital were significantly different between the two groups.

**Table 2:** Baseline characteristics of patients with *rt‐PA*

Characteristic	Total	Phase 1	Phase 2	*p*‐value
Patients included (%)		501 (41.5)	706 (58.5)	
Age (mean ± *SD*)	63.73 ± 12.34	63.04 ± 12.42	64.22 ± 12.09	.097
Male	834 (69.1)	349 (69.7)	485 (68.7)	.721
Hypertension	736 (61.0)	296 (59.1)	440 (62.3)	.255
Diabetes	277 (22.9)	108 (21.6)	169 (23.9)	.332
Hyperlipidemia	81 (6.7)	31 (6.2)	50 (7.1)	.541
Coronary heart disease	111 (9.2)	41 (8.2)	70 (9.9)	.305
Atrial fibrillation	113 (9.4)	44 (8.8)	69 (9.8)	.560
History of cerebral infarction	173 (14.3)	87 (17.4)	86 (12.2)	.011
Admission NIHSS score (mean ± *SD*)	8.30 ± 7.11	7.97 ± 6.95	8.53 ± 7.22	.182
EMS use	417 (34.5)	147 (29.3)	270 (38.2)	.001
Arrival hospital				
PSC	368 (30.5)	122 (24.4)	246 (34.8)	.000
CSC	839 (69.5)	379 (75.6)	460 (65.2)	

Note

The *p*‐values are based on Student's *t* test (normal distribution) and chi‐square test (categorical variables).

Abbreviations: CSC, comprehensive stroke center; EMS, emergency medical service; NIHSS, National Institutes of Health Stroke Scale; PSC, primary stroke center; rt‐PA, recombinant tissue plasminogen activator.

The median onset‐to‐door time decreased from 118 min (IQR: 75–160 min) in phase 1 to 105 min (IQR: 68–150 min; *p* = .002) in phase 2. Furthermore, 1,207 (67.6%) patients were treated with intravenous rt‐PA, which is increase from 63.9% in phase 1 to 70.5% in phase 2. Among them, the onset‐to‐door time and onset‐to‐needle time were reduced by five minutes (100 [IQR: 62–135] vs. 105 [IQR: 70–145], *p* = .005) and nine minutes (158 [IQR: 124–197] vs. 167 [IQR: 132–214], *p* = .001), respectively. However, the door‐to‐needle time was not observed to be significantly shorter in phase 2 (*p* = .312) (Table 3).

**Table 3:** Timeline comparison between two groups

Characteristic	Eligible patients	Phase 1	Phase 2	*p*‐value
Onset‐to‐door time (min)	1,769	118 (75–160)	105 (68–150)	.002
No. of patients with rt‐PA	1,207	501 (63.9)	706 (70.5)	.003
Onset‐to‐door time with rt‐PA (min)	1,190	105 (70–145)	100 (62–135)	.005
Door‐to‐needle time with rt‐PA (min)	1,207	52 (38–76)	52 (38–70)	.312
Onset‐to‐needle time with rt‐PA (min)	1,190	167 (132–214)	158 (124–197)	.001

Note

Values are expressed as a number (%) or median value (interquartile range). The Mann–Whitney *U* test was used for comparison of timeline between two phases. Abbreviation: rt‐PA, recombinant tissue plasminogen activator.

We performed a further comparison of patients treated with rt‐PA between the two phases according to the time stratification. The proportion of patients with an onset‐to‐door time ≤ 2 hr and ≤ 3.5 hr among phase 2 was higher than in phase 1 (82.9% vs. 77.4%, *p* = .019; 97.6% vs. 93.6%, *p* = .001). Similar results were observed for onset‐to‐needle time ≤ 2.5 hr, ≤ 3 hr, and ≤ 3.5 hr (45.2% vs. 38.5%, *p* = .021; 66.0% vs. 60.3%, *p* = .042; 81.2% vs. 72.7%, *p* < .001), respectively (Table 4).

**Table 4:** Timeline and outcome comparison of patients treated with rt‐PA in the two phases

Variable	Total	Phase 1	Phase 2	*p* value
Onset‐to‐door time ≤ 2 hr	973 (80.6)	388 (77.4)	585 (82.9)	.019
Onset‐to‐door time ≤ 3.5 hr	1,158 (95.9)	469 (93.6)	689 (97.6)	.001
Door‐to‐needle time ≤ 0.5 hr	171 (14.2)	78 (15.6)	93 (13.2)	.240
Door‐to‐needle time ≤ 1 hr	792 (65.6)	315 (62.9)	477 (67.6)	.091
Onset‐to‐needle time ≤ 2.5 hr	512 (42.4)	193 (38.5)	319 (45.2)	.021
Onset‐to‐needle time ≤ 3 hr	768 (63.6)	302 (60.3)	466 (66.0)	.042
Onset‐to‐needle time ≤ 3.5 hr	937 (77.6)	364 (72.7)	573 (81.2)	<.001

Abbreviation: rt‐PA, recombinant tissue plasminogen activator.

Additionally, we observed the timelines for rt‐PA patients with and without EMS usage. The onset‐to‐door time and onset‐to‐needle time were shorter for patients who were EMS‐transported (*p* < .001); however, we did not observe similar superiority of EMS transportation in the door‐to‐needle time (*p* = .763). The patients with atrial fibrillation and higher baseline NIHSS scores and who were included in phase 2 were more likely to use EMS (Tables S2 and S3).

**Figure 2:**
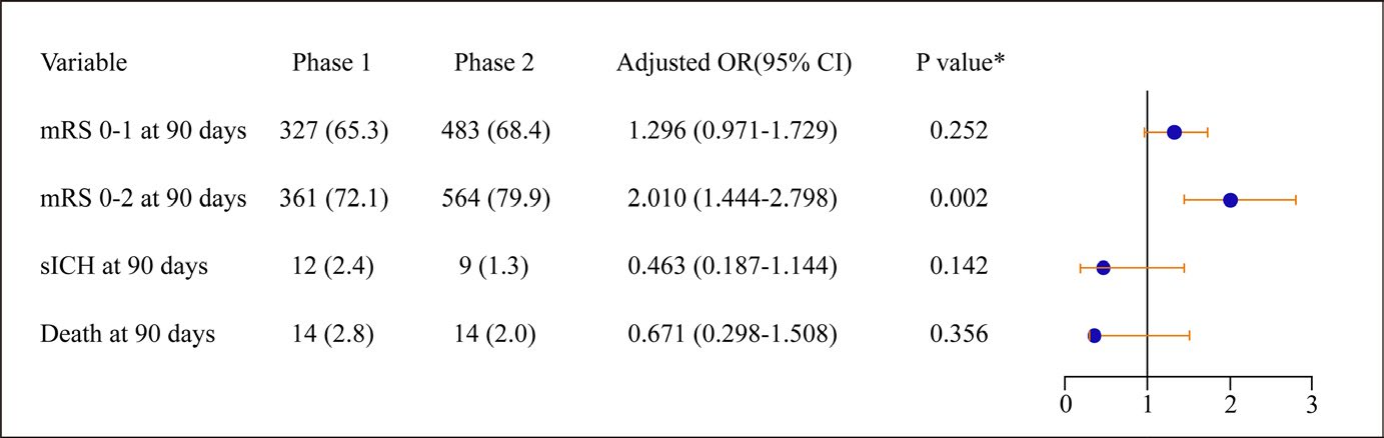
Outcome comparison of patients treated with *rt‐PA* in two phases. mRS, modified Rankin Scale; rt‐PA, recombinant tissue plasminogen activator; sICH, symptomatic intracranial hemorrhage

We documented higher rates of independent functional outcome following rt‐PA in phase 2 compared to phase 1 (79.9% vs. 72.1%, *p* = .002). The rate of favorable clinical outcome at 90 days was slightly, but not significantly better in phase 2 than in phase 1 (68.4% vs. 65.3%, *p* = .252). No differences in the rates of sICHs (1.3% vs. 2.1%, *p* = .142) and mortality at 90 days (2.0% vs. 2.8%, *p* = .356) were observed between the two phases (Figure 2). The percentages of patients with mRS scores at 90 days are presented in Figure S1, and after adjustment for age, sex, admission NIHSS scores, the mode of transportation, and the type of arrived hospital, the significance persisted (mRS score 0–2 at 90 days, adjusted odds ratio, 2.010; 95% CI, 1.444–2.798).

Among the 925 patients who achieved an independent outcome at 90 days, age, gender, diabetes, coronary heart disease, atrial fibrillation, history of cerebral infarction, admission NIHSS score, transportation means, onset‐to‐needle time, and period of presentation were significantly related to 90‐day functional independence in the initial univariable analysis. In the multivariable regression analysis, young in age (OR: 1.811; 95% CI: 1.304–2.516; *p* < .001), lower admission NIHSS score (OR: 39.120; 95% CI: 23.477–65.188; *p* < .001 and OR: 18.324; 95% CI: 11.425–29.388; *p* < .001 and OR: 3.123; 95% CI: 2.044–4.773; *p* < .001), shorter onset‐to‐needle time (OR: 0.994; 95% CI: 0.992–0.997; *p* < .001), and treatment with rt‐PA in phase 2 (OR: 1.965; 95% CI: 1.413–2.731; *p* < .001) were identified as independent predictors of 90‐days mRS 0–2 (Table 5).

**Table 5:** Univariable and multivariable regression analyses of the independent outcomes

Variable	Univariable logistic regression analysis		Multivariable logistic regression analysis	
	OR (95% CI)	*p* value^a^	OR (95% CI)	*p* value
Age (Ref: ≥65)				
<65	2.298 (1.843–2.865)	<.001	1.811 (1.304–2.516)	<.001
Male/female	0.660 (0.499–0.873)	.004	0.951 (0.674–1.341)	.773
Hypertension	0.942 (0.716–1.240)	.671		
Diabetes	0.771 (0.567–1.048)	.097	0.752 (0.524–1.081)	.124
Hyperlipidemia	1.159 (0.667–2.013)	.601		
Coronary heart disease	0.662 (0.432–1.016)	.059	1.040 (0.607–1.780)	.888
Atrial fibrillation	0.455 (0.303–0.683)	.000	1.010 (0.615–1.660)	.967
History of cerebral infarction	0.584 (0.411–0.830)	.003	0.694 (0.450–1.068)	.097
Admission NIHSS score （Ref: ≥15）				
0–4	46.100 (31.107–68.320)	<.001	39.120 (23.477–65.188)	<.001
5–9	14.983 (10.385–21.617)	<.001	18.324 (11.425–29.388)	<.001
10–14	3.379 (2.346–4.866)	<.001	3.123 (2.044–4.773)	<.001
EMS usage	0.496 (0.378–0.652)	<.001	1.097 (0.781–1.541)	.592
CSC/PSC	1.280 (0.950–1.724)	.105		
Onset‐to‐needle time	0.998 (0.995–1.000)	.064	0.994 (0.992–0.997)	<.001
Phase 2	1.540 (1.178–2.014)	.002	1.965 (1.413–2.731)	<.001

Abbreviations: CSC, comprehensive stroke center;EMS, emergency medical service; NIHSS, National Institutes of Health Stroke Scale; PSC, primary stroke center.

^a^ Cut‐off of *p* < .1 was used for selection of candidate variables for inclusion in multivariable logistic regression models.

## 4 DISCUSSION

The Chinese stroke emergency map not only provides patients with the locations of nearby available thrombolytic hospitals, but also a quality management system, from early stroke recognition, timely arrival, and rapid treatment to minimize the prehospital and in‐hospital delays for thrombolysis. In our study, the significance of the implementation of the *SEM* is that the proportion of patients who arrived at hospitals within a 4.5‐hr time window and received rt‐PA increased. The median onset‐to‐door time was reduced from 118 min in phase 1 to 105 min in phase 2. Furthermore, the onset‐to‐door time and onset‐to‐needle time were reduced by five minutes and nine minutes for patients treated with rt‐PA, respectively. Although the door‐to‐needle time was not shortened significantly in phase 2, the proportion of patients with DNT ≤ 1 hr increased from 62.9% to 67.6%. Moreover, decreased onset‐to‐needle time was independently related to the independent functional outcome at 90 days, without increased rates of hemorrhagic complications or mortality. This phenomenon is likely due to the promotion of *SEM*, effectively increasing the timely recognition of stroke and awareness of intravenous thrombolysis, all while guiding more patients to the closest qualified hospitals, effectively avoiding the prehospital delays previously caused by referral systems (Huang et al., 2015).

Our findings demonstrate that the mean DNT was 52 min, which is consistent with the international recommendation of DNT < 60 min (Jauch et al., 2013). However, there is still a large gap compared with more developed countries. In the Helsinki Stroke Thrombolysis Registry, the median DNT has been cut to 20 min, 94% of patients were treated within 60 min of arrival (Meretoja et al., 2012), and the Canadian guidelines recommend a 30‐min median DNT target for patients treated with rt‐PA (Casaubon et al., 2015). These differences may be due to multiple factors. The majority of patients in our study were self‐transported (64.1%), which to some extent can reduce the delay in onset call EMS arrival time, but it may also fail to ensure timely activation of the in‐hospital emergency management program for stroke, which is triggered by referral or triage in the emergency room.

Several previous papers have reported the beneficial effects of the EMS on the reduction of the prehospital pathway and an increase in the rate of thrombolytic treatment by prehospital hospital notification (Gu et al., 2019). However, here, the use of EMS was slightly increased in phase 2. This corrective factor did not improve in‐hospital delay in our study, possibly because only 35.9% of stroke patients in the time window were transferred to the hospital by EMS, which was much lower than the 63.7% observed in the “Get With The Guidelines ― Stroke” program (Ekundayo et al., 2013). A survey of three cities in China indicated that only 18.8% of residents called for emergency services (Jiang et al., 2016). This is a reminder that future work should focus on culture‐adapted stroke education, and recent “stroke‐1‐2‐0” or “FAST” public awareness campaigns are likely to promote awareness of stroke and the EMS activation. Additionally, we observed that patients with atrial fibrillation or greater stroke severity were more likely to use the EMS. For these patients, the neurologist evaluation (Vahidy, Rahbar, Lal, Grotta, & Savitz, 2015) and the patients’ caregivers’ concerns about the risk of thrombolysis may cause delays for treatment. Standardized training has been implemented to teach clinicians to be more persuasive when educating patients and caregivers regarding the importance of timely intervention for stroke.

The clinical outcome benefit associated with decreased time to treatment with rt‐PA and without increased risk of intracranial hemorrhage and death (Fonarow et al., 2014; Lees et al., 2010). Our findings are similar to those of previous research. In the “Get with The Guidelines ― Stroke” registry, the patients treated with rt‐PA within 60 min were more likely to be discharged to their home and capable of independent ambulation at discharge than the patients treated within 61–270 min (Tsivgoulis et al., 2017). These findings indicate that intensive efforts to improve the timeliness of thrombolytic therapy are vital. The EMS rapid arrival on the scene, prenotification, and timely activation of the emergency services are important to improve the clinical outcomes.

Due to a poor understanding of stroke, patients typically prefer to select the CSCs that are representative of the relatively high‐level hospitals. However, the unequal geographical distribution of CSCs and PSCs results in limited access to thrombolysis therapy for some regions. Most CSCs are located in densely populated and congested areas with numerous self‐transport patients in emergency departments, and wait times for triage are extensive. This is another possible explanation for in‐hospital delays. In addition, the prevalence of stroke in rural areas is higher than in urban areas. Therefore, the other significance of promoting the *SEM* is that it results in training more PSCs located in rural areas eligible for thrombolysis. Patients choosing a qualified PSC might be a better option for expedited treatment. Although the proportion of patients transferred to PSCs has increased since the promotion of the SEMs, it is still significantly lower than those transported to CSCs. Therefore, actively correcting misconceptions, utilizing the emergency service system to select an optimal stroke center, and establishing a proper referral system are likely to benefit stroke patients.

### 4.1 Limitations

Our study has several limitations. First, the study data were based on self‐reports from every qualified hospital included in the *SEM*, and therefore, some missing data were unavoidable as patients of nonqualified hospitals within the time window were not recorded. Second, this is a retrospective observational study, and because the data were not primarily collected for research, there may be some gaps in the dataset, such as the time from the EMS system receipt of the call to arrival on the scene, the time of EMS alerting, the time of door to CT, and the time of door to laboratory. Hence, we could not effectively assess potential reasons for the observation of no significant reduction in DNT. Third, the outcomes at 90 days for patients treated with EVT alone were not documented in this study and should be included in future studies. Fourth, due to the lack of unified management, stroke patients within a 4.5‐hr time window were not collected before the *SEM* was developed, we failed to compare the deadlines and function outcome before and after the *SEM* implementation. Finally, the *SEM* protocol did not have a unified standard process of emergency management program in hospitals, and clinical outcomes may be affected by factors other than delays to treatment. To address these issues, further work is necessary to establish the network platform of the stroke emergency map, develop standardized case descriptions and timeline records, and maximize the facilitation of the stroke‐related health service system based on *SEM*.

## 5 CONCLUSIONS

In conclusion, the implementation of the stroke emergency map is more likely to reduce the prehospital delay and improve the likelihood of function outcomes. Future efforts are necessary to encourage other thrombolysis capable hospitals to participate in the *SEM* alliance, continually improve public awareness of stroke, and increase the usage of EMS.

## CONFLICTS OF INTEREST

We have no conflicts of interest to declare.

## AUTHOR CONTRIBUTIONS

WL contributed to conceive and revise the manuscript. TZ performed the data collection, and wrote and revised the manuscript. XZ designed the study and revised the manuscript. TZ and XZ are co‐correspondence authors. HS contributed to the conception and design of study. FZ and SL contributed to perform the analysis with constructive discussions. HS, ZZ, NZ, and JS performed the data collection and analysis.

## ETHICAL APPROVAL

The ethics committee of the Taiyuan Central Hospital, Shanxi, China, approved the study protocol. Patient consent was waived by the ethics committee of the Taiyuan Central Hospital due to the retrospective design of the study.

### Peer Review

The peer review history for this article is available at https://publons.com/publon/10.1002/brb3.1743.

## Supporting information

Fig S1

Table S1‐S3

## Data Availability

The datasets used during this study are available from the corresponding author on reasonable request.
