# Supplementary figures and images for: Improving timely treatment with a stroke emergency map: The case of northern China
Source: Brain Behav. 2020 Jul 11;10(8):e01743. doi: 10.1002/brb3.1743 (PMC7428498; doi:10.1002/brb3.1743)

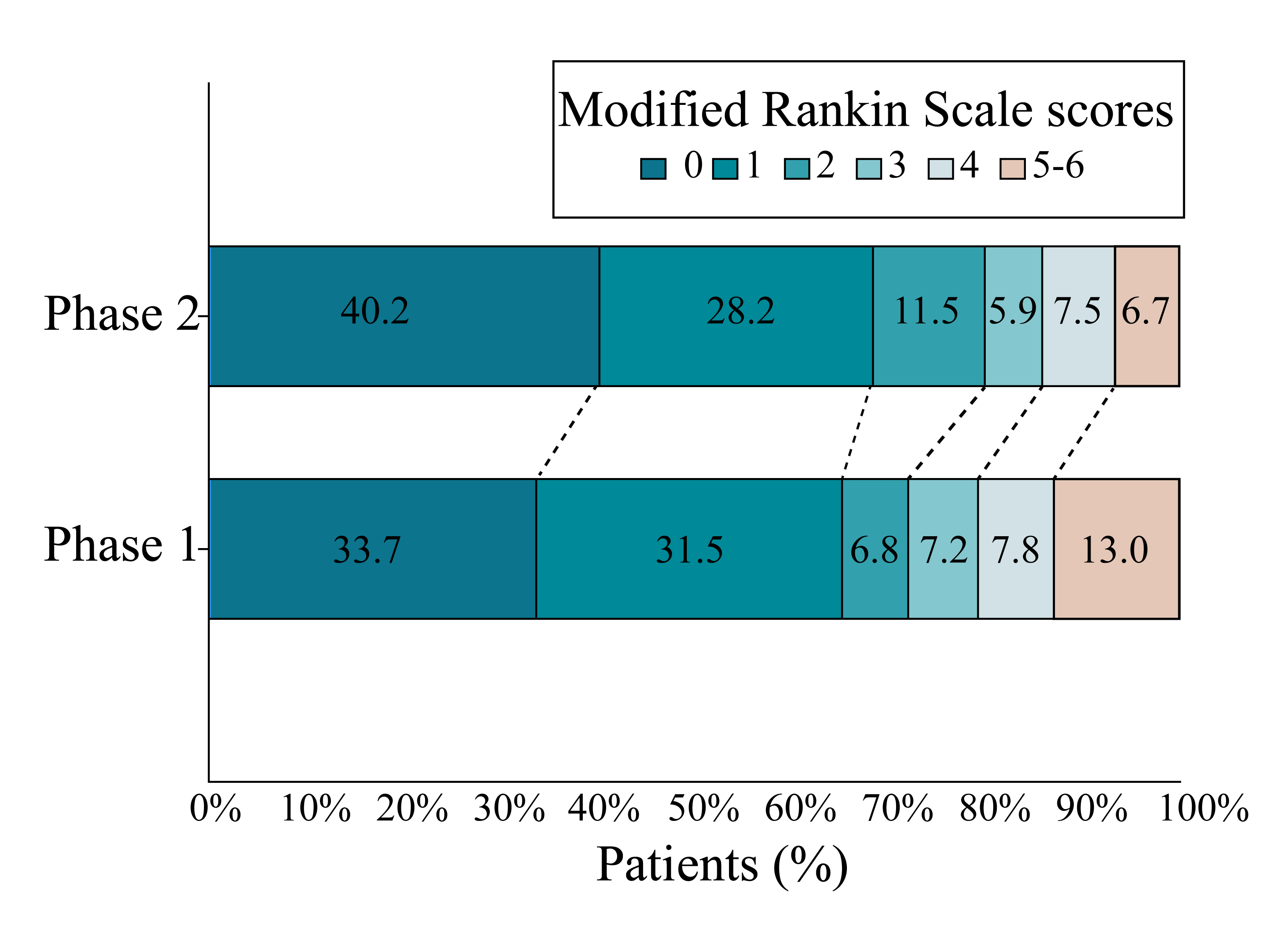

Supplement: Supplementary file 1 — Fig S1 [file BRB3-10-e01743-s001.tif]
