# Supplementary material for: Improving timely treatment with a stroke emergency map: The case of northern China
Source: Brain Behav. 2020 Jul 11;10(8):e01743. doi: 10.1002/brb3.1743 (PMC7428498; doi:10.1002/brb3.1743)
Supplement: Supplementary file 2 — Table S1‐S3 [file BRB3-10-e01743-s002.docx]

Supplement table 1. Baseline characteristics of patients within the initial 15 hospitals between two phases

| Characteristic | Total | Phase1 | Phase 2 | *P*-Value |
| --- | --- | --- | --- | --- |
| Patients included (%) | 1724 | 784 (45.5) | 940 (54.5) |  |
| Age (mean ± SD) | 64.30±12.61 | 63.97±12.72 | 64.58±12.51 | 0.324 |
| Male | 1192 (69.1) | 546 (69.6) | 646 (68.7) | 0.681 |
| Hypertension | 1041 (60.4) | 456 (58.2) | 585 (62.2) | 0.085 |
| Diabetes | 426 (24.7) | 199 (25.4) | 227 (24.1) | 0.554 |
| Hyperlipidemia | 142 (8.2) | 74 (9.4) | 68 (7.2) | 0.097 |
| Coronary heart disease | 152 (8.8) | 61 (7.8) | 91 (9.7) | 0.166 |
| Atrial fibrillation | 154 (8.9) | 66 (8.4) | 88 (9.4) | 0.494 |
| History of cerebral infarction | 239 (13.9) | 118 (15.1) | 121 (12.9) | 0.192 |
| Admission NIHSS score, (mean ± SD) | 7.82±7.67 | 7.63±7.46 | 7.99±7.84 | 0.329 |
| EMS use | 606 (35.2) | 254 (32.4) | 352 (37.4) | 0.029 |
| Type of treatment |  |  |  |  |
| Only intravenous rt-PA | 1046 (60.7) | 454 (57.9) | 592 (63.0) | 0.074 |
| Intravenous rt-PA+EVT | 109 (6.3) | 47 (6.0) | 62 (6.6) |  |
| Only EVT | 71 (4.0) | 32 (4.1) | 39 (4.1) |  |
| Other | 498 (28.9) | 251 (32.0) | 247 (26.3) |  |
| Arrival hospital |  |  |  |  |
| PSC | 416 (24.1) | 172 (21.9) | 244 (26.0) | 0.052 |
| CSC | 1308 (75.9) | 612 (78.1) | 696 (74.0) |  |

Footnotes: The p-values are based on the Student’s t-test (normal distribution),the χ^2^ test (categorical variables). Abbreviations: *NIHSS* National Institutes of Health Stroke Scale, *rt-PA* Recombinant tissue plasminogen activator, *EVT* Endovascular treatment, *EMS* Emergency medical service, *PSC* Primary stroke center, *CSC* Comprehensive stroke center

Supplement Table 2. Characteristics of the EMS use and no EMS use groups in patients with rt-PA

| Variable | EMS use  (n=417, 34.5%) | No EMS use (n=790, 65.5%) | *P* Value |
| --- | --- | --- | --- |
| Age (mean ± SD) | 65.93±11.885 | 62.57±12.268 | ＜0.001 |
| Male | 280 (67.1) | 554 (70.1) | 0.287 |
| Hypertension | 266 (63.8) | 470 (59.5) | 0.146 |
| Diabetes | 104 (24.9) | 173 (21.9) | 0.232 |
| Hyperlipidemia | 29 (7.0) | 52(6.6) | 0.806 |
| Coronary heart disease | 53 (12.7) | 58 (7.3) | 0.002 |
| Atrial fibrillation | 65 (15.6) | 48 (6.1) | ＜0.001 |
| History of cerebral infarction | 69 (16.5) | 104 (13.2) | 0.111 |
| Admission NIHSS score, (mean ± SD) | 11.06±7.87 | 6.84±6.21 | ＜0.001 |
| Arrival hospital |  |  |  |
| PSC | 124 (29.7) | 244 (30.9) | 0.680 |
| CSC | 293 (70.3) | 546 (69.1) |  |
| Timeline Measure |  |  |  |
| Onset-to-door time with rt-PA (min) | 89 (57-120) | 110 (70-150) | ＜0.001 |
| Door-to-needle time with rt-PA ( min) | 51 (37.5-76) | 52 (39-71) | 0.763 |
| Onset-to-needle time with rt-PA (min) | 153 (119-192) | 166 (132-207) | ＜0.001 |
| Onset to door time ≤ 2h | 371 (89.0) | 602 (76.2) | 0.000 |
| Onset to door time ≤ 3.5h | 410 (98.3) | 748 (94.7) | 0.002 |
| Door to needle time ≤ 0.5h | 56 (13.4) | 115 (14.6) | 0.593 |
| Door to needle time ≤ 1h | 265 (63.5) | 527 (66.7) | 0.272 |
| Treatment with rt-PA by 2.5h | 205 (49.2) | 307 (38.9) | 0.001 |
| Treatment with rt-PA by 3h | 294 (70.5) | 474 (60.0) | 0.000 |
| Treatment with rt-PA by 3.5h | 341 (81.8) | 596 (75.4) | 0.012 |

Footnotes: The p-values are based on the Student’s t-test (normal distribution),the χ^2^ test (categorical variables). Abbreviations: *NIHSS* National Institutes of Health Stroke Scale, *rt-PA* Recombinant tissue plasminogen activator, *EMS* Emergency medical service, *PSC* Primary stroke center, *CSC* Comprehensive stroke center

Supplement table 3. Univariate and multivariable regression analyses of emergency medical service (EMS) usage.

| Variable | Univariable logistic regression analysis | | Multivariable logistic regression analysis | |
| --- | --- | --- | --- | --- |
|  | OR (95% CI) | *P* value^*^ | OR (95% CI) | *P* value |
| Age (Ref: ≥65) |  |  |  |  |
| <65 | 0..689 (0.567-0.837) | <0.001 | 0..868 (0.705-1.070) | 0.186 |
| Men/Women | 1.149 (0.890-1.482) | 0.287 |  |  |
| Hypertension | 1.199 (0.939-1.532) | 0.146 |  |  |
| Diabetes | 1.185 (0.897-1.566) | 0.232 |  |  |
| Hyperlipidemia | 1.061 (0.663-1.698) | 0.806 |  |  |
| Coronary heart disease | 1.838 (1.240-2.723) | 0.002 | 1.205 (0.844-1.721) | 0.306 |
| Atrial fibrillation | 2.855 (1.925-4.233) | <0.001 | 1.611 (1.128-2.300) | 0.009 |
| History of cerebral infarction | 1.308 (0.940-1.820) | 0.111 |  |  |
| Admission NIHSS score （Ref: ≥15） |  |  | 1.078 (1.058-1.098) | 0.000 |
| 0-4 | 0.185 (0.139-0.247) | <0.001 | 0.210 (0.156-0.284) | <0.001 |
| 5-9 | 0.294 (0.216-0.402) | <0.001 | 0.325 (0.236-0.446) | <0.001 |
| 10-14 | 0.657 (0.466-0.928) | 0.017 | 0.702 (0.494-0.996) | 0.048 |
| PSC | 0.947 (0.731-1.226) | 0.680 |  |  |
| Phase 2 | 1.491 (1.167-1.905) | 0.001 | 1.275(1.036-1.568) | 0.022 |

^*^Cut-off of *P* < 0.1 was used for selection of candidate variables for inclusion in multivariable logistic regression models. Abbreviations: *PSC* Primary stroke center, *CI* Confidence interval, *OR* Odds ratios.

Supplement figure. Distribution of modified Rankin scale scores at 90 days. Patients treated with rt-PA in phase 2 achieved relatively better independent clinical outcomes than those in phase (mRS score 0–2 at 90 days, 79.9% versus 72.1%, *P*=0.002).
